# Survival outcome and prognostic factors for early-onset and late-onset metastatic colorectal cancer: a population based study from SEER database

**DOI:** 10.1038/s41598-024-54972-3

**Published:** 2024-02-22

**Authors:** Bingyi Ren, Yichen Yang, Yi Lv, Kang Liu

**Affiliations:** 1 Department of Hepatobiliary Surgery, First Affiliated Hospital of Xi’an Jiaotong University, Xi’an, 710061 Shaanxi Province China; 2 National Local Joint Engineering Research Center for Precision Surgery and Regenerative Medicine, First Affiliated Hospital of Xi’an Jiaotong University, Xi’an, 710061 Shaanxi Province China

**Keywords:** Early-onset colorectal cancer, Metastatic cancer, Survival, Cancer, Gastrointestinal cancer, Metastasis

## Abstract

Colorectal cancer is the third most common cancer worldwide and there has been a concerning increase in the incidence rate of colorectal cancer among individuals under the age of 50. This study compared the survival outcome between early-onset and late-onset metastatic colorectal cancer to find the differences and identify their prognostic factors. We obtained patient data from SEER database. Survival outcome was estimated using Kaplan–Meier survival curves and compared using the log-rank test. Univariate and multivariate analyses were conducted utilizing COX models to identify their independent prognostic factors. A total of 10,036 early-onset metastatic colorectal (EOCRC) cancer patients and 56,225 late-onset metastatic colorectal cancer (LOCRC) patients between 2010 and 2019 were included in this study. EOCRC has more survival benefits than LOCRC. Tumor primary location (*p* < 0.001), the location of metastasis (*p* < 0.001) and treatment modalities (*p* < 0.001) affect the survival outcomes between these two groups of patients. Female patients had better survival outcomes in EOCRC group (*p* < 0.001), but no difference was found in LOCRC group (*p* = 0.57). In conclusion, our study demonstrated that EOCRC patients have longer survival time than LOCRC patients. The sex differences in survival of metastatic colorectal cancer patients are associated with patients’ age. These findings contribute to a better understanding of the differences between metastatic EOCRC and LOCRC, and can help inform the development of more precise treatment guidelines to improve prognosis.

## Introduction

Colorectal cancer is the third most common cancer worldwide and the second leading cause of cancer death in the US^1,2^. While the overall incidence rate of colorectal cancer is declining due to the emphasis on prevention, there has been a concerning increase in the incidence rate of colorectal cancer among individuals under the age of 50 since the 1990s^3^. This subset of patients is referred to as early-onset colorectal cancer^4^. In certain countries, the rate of early-onset colorectal cancer is growing at an alarming rate of 2–3%. It is projected that by 2030, early-onset colorectal cancer will surpass other cancers to become the most common cancer among individuals aged 20–49 in the United States^5,6^.

Not only the proportion of early-onset colorectal cancer patients is increasing, but early-onset colorectal cancer has more aggressive nature and higher likelihood of metastasis compared to late-onset colorectal cancer^7–9^. Metastasis significantly impacts patient prognosis^10^, making it crucial for researchers to focus on patients with metastatic early-onset colorectal cancer. However, there is controversy on the prognosis of metastatic early-onset colorectal cancer. While some multicenter studies suggest no difference in prognosis between metastatic early-onset colorectal cancer and late-onset colorectal cancer^11,12^, other studies indicate a disparity between the two^13,14^. Therefore, further research is necessary to explore this direction, and multiple data sources are required to validate these findings.

In this study, we retrieved the colorectal cancer population from the SEER database to make comparisons between early-onset and late-onset metastatic colorectal cancer and figure out the possible prognostic factors.

## Methods

### Data sources

The Surveillance, Epidemiology, and End Results (SEER) is a public cancer database created by the National Cancer Institute (NCI), which includes 18 population-based tumor registries covering approximately 28% of the Americans^15^. The data in this study were obtained from the SEER Program SEER*Stat software released in November 2021 (version8.4.0). According to the definition, a total of 66,261 patients of CRC registered between 2010 and 2019 were screened and divided into early-onset colorectal cancer (EOCRC) (N = 10,036) and late-onset colorectal cancer (LOCRC) (N = 56,225). EOCRC was defined as CRC arising in adults 20–49 years of age, while LOCRC was defined as CRC arising in adults 50–75 years of age^16^.

**Figure 1:**
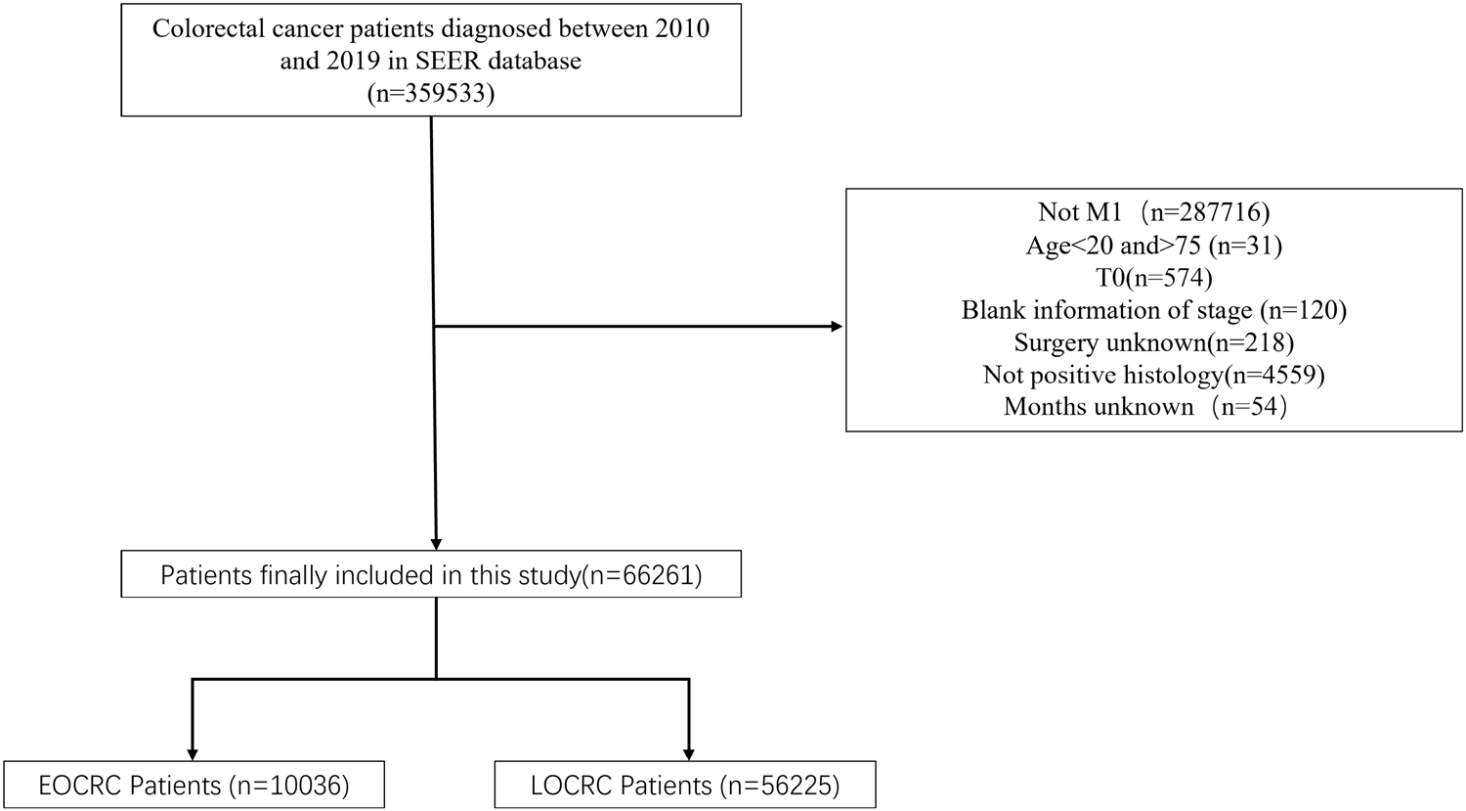
Flow chart illustrating patients selection.

Patient selection criteria were as follows: (1) colorectal cancer patients diagnosed between 2010 and 2019; (2) exclusion of patients not in M1 stage; (3) exclusion of patients younger than 20 years old or older than 75 years old; (4) exclusion of patients in T0 stage; (5) exclusion of patients with blank information of stage; (6) exclusion of patients whose surgery information is unknown; (7) exclusion of patients whose histology is not positive (8). exclusion of patients with unknown survival months. A flow chart of the study population selection was displayed in Fig. 1.

### Data collection

The following variables were selected for this study: year of diagnosis, gender, race, primary site, histological type, T stage, N stage, grade, surgery, radiotherapy, chemotherapy, CEA, marital status and metastatic sites.

### Statistical analysis

All statistical analyses were implemented with R (version 4.1.2) software. Statistical significance was set at *p* < 0.05. Baseline characteristics according to EOCRC and LOCRC groups were compared using test for categorical variables. OS and CSS were calculated based on the Kaplan–Meier curves and compared between EOCRC versus LOCRC patients using the log-rank χ^2^test^17^. Overall survival (OS) were the primary endpoints of this study. The overall survival (OS) refers to the time passed from the time of the initial diagnosis of CRC to the time of death from any cause. Univariate and multivariate analyses were conducted utilizing COX models to reveal the independent prognostic factors for EOCRC and LOCRC^18^. Hazard ratios (HRs) and 95% confidence interval (CI) calculations were based on COX models.

## Results

### Baseline characteristics

In this study, 359,533 patients diagnosed with colorectal cancer between 2010 and 2019 were extracted from the SEER database, of which 66,261 met our screening criteria for further study. Among them, there were 10,036 patients with early-onset colorectal cancer and 56,225 patients with late-onset colorectal cancer (Table 1).

**Table 1:** Patient characteristics in the study.

Variable	EOCRC	LOCRC	*p*
Gender			0.019
Male	5368 (53.5%)	30,791 (54.8%)	
Female	4668 (46.5%)	25,434 (45.2%)	
Year of diagnosis			0.020
2010	897 (8.94%)	5340 (9.50%)	
2011	925 (9.22%)	5419 (9.64%)	
2012	904 (9.01%)	5366 (9.54%)	
2013	988 (9.84%)	5343 (9.50%)	
2014	982 (9.78%)	5698 (10.1%)	
2015	976 (9.72%)	5608 (9.97%)	
2016	1067 (10.6%)	5768 (10.3%)	
2017	1122 (11.2%)	5725 (10.2%)	
2018	1065 (10.6%)	5905 (10.5%)	
2019	1110 (11.1%)	6053 (10.8%)	
Race			< 0.001
White	7351 (73.2%)	43,306 (77.0%)	
Black	1486 (14.8%)	7718 (13.7%)	
American Indian	119 (1.19%)	491 (0.87%)	
Asian	1028 (10.2%)	4556 (8.10%)	
Unknown	52 (0.52%)	154 (0.27%)	
Primary site			< 0.001
Right-sided	2177 (21.7%)	19,307 (34.3%)	
Left-sided	3204 (31.9%)	13,827 (24.6%)	
Rectum	2405 (24.0%)	10,479 (18.6%)	
Others	2250 (22.4%)	12,612 (22.4%)	
Histological type			< 0.001
Adenocarcinoma	9535 (95.0%)	52,685 (93.7%)	
Others	501 (4.99%)	3540 (6.30%)	
T			< 0.001
T1	697 (6.94%)	4307 (7.66%)	
T2	245 (2.44%)	1199 (2.13%)	
T3	3180 (31.7%)	16,160 (28.7%)	
T4	3132 (31.2%)	15,816 (28.1%)	
Unknown	2782 (27.7%)	18,743 (33.3%)	
N			< 0.001
N0	2715 (27.1%)	18,135 (32.3%)	
N1	3080 (30.7%)	15,429 (27.4%)	
N2	2553 (25.4%)	11,924 (21.2%)	
Unknown	1688 (16.8%)	10,737 (19.1%)	
Grade			< 0.001
1/2	5674 (56.5%)	29,314 (52.1%)	
3/4	2245 (22.4%)	12,357 (22.0%)	
Unknown	2117 (21.1%)	14,554 (25.9%)	
Surgery			< 0.001
No	4367 (43.5%)	28,521 (50.7%)	
Yes	5669 (56.5%)	27,704 (49.3%)	
Radiation			< 0.001
No	8339 (83.1%)	49,786 (88.5%)	
Yes	1697 (16.9%)	6439 (11.5%)	
Chemotherapy			0.000
No	1486 (14.8%)	20,939 (37.2%)	
Yes	8550 (85.2%)	35,286 (62.8%)	
CEA			< 0.001
Negative/Borderline	1503 (15.0%)	6891 (12.3%)	
Positive	5907 (58.9%)	31,239 (55.6%)	
Unknown	2626 (26.2%)	18,095 (32.2%)	
Marital status			0.000
Married	5236 (52.2%)	27,871 (49.6%)	
Single/unmarried	3309 (33.0%)	10,412 (18.5%)	
Divorced/widowed/separated	1019 (10.2%)	15,154 (27.0%)	
Unknown	472 (4.70%)	2788 (4.96%)	
Bone metastases			0.002
No	9216 (91.8%)	51,121 (90.9%)	
Yes	568 (5.66%)	3343 (5.95%)	
Unknown	252 (2.51%)	1761 (3.13%)	
Brain metastases			< 0.001
No	9675 (96.4%)	53,580 (95.3%)	
Yes	102 (1.02%)	753 (1.34%)	
Unknown	259 (2.58%)	1892 (3.37%)	
Liver metastases			0.011
No	2934 (29.2%)	15,972 (28.4%)	
Yes	6972 (69.5%)	39,324 (69.9%)	
Unknown	130 (1.30%)	929 (1.65%)	
Lung metastases			< 0.001
No	7657 (76.3%)	40,667 (72.3%)	
Yes	2102 (20.9%)	13,735 (24.4%)	
Unknown	277 (2.76%)	1823 (3.24%)	

### Comparison of overall survival between EOCRC and LOCRC

**Figure 2:**
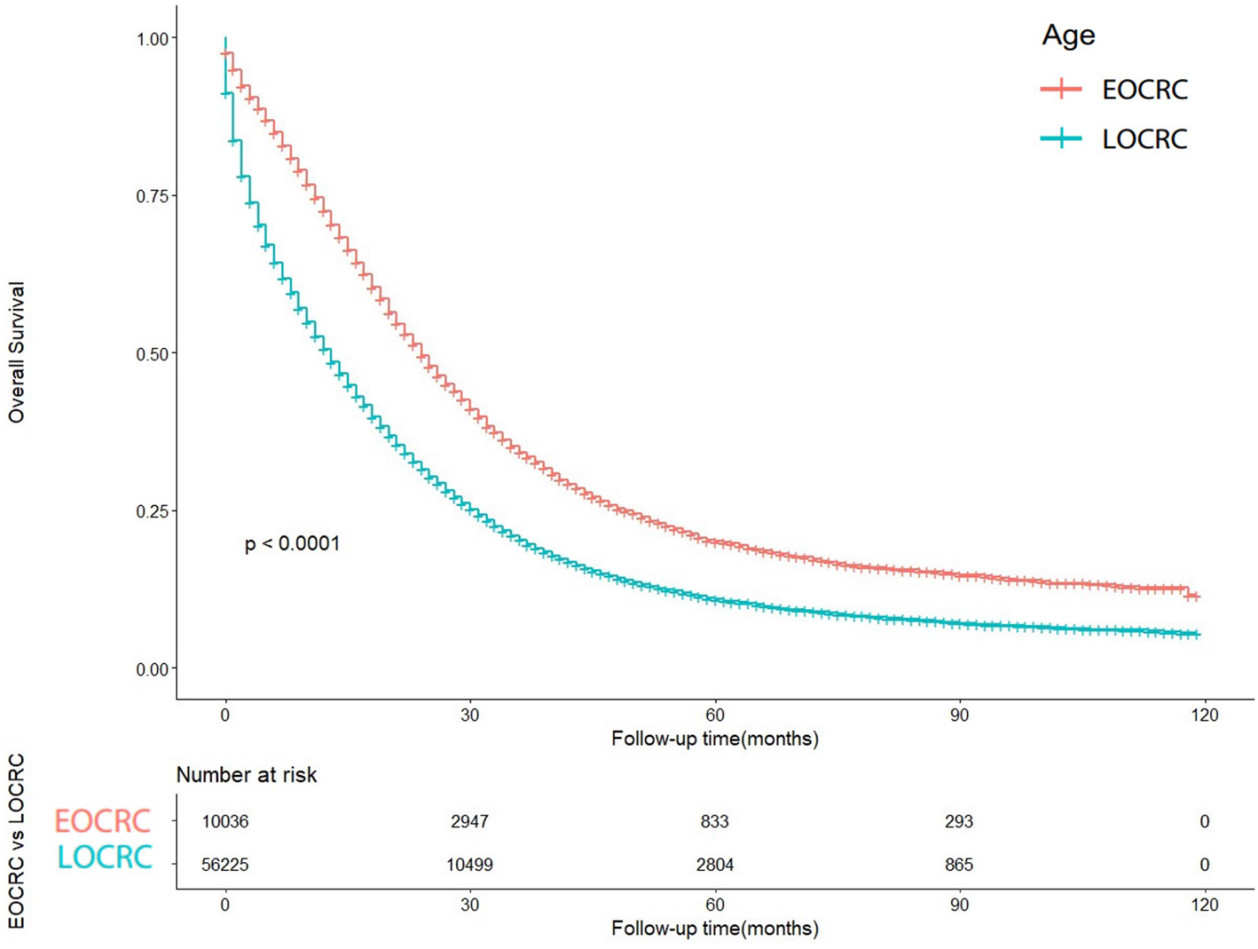
Kaplan–Meier-curves for overall survival in EOCRC patients and LOCRC patients. Life tables for patients at risk are given below the plot.

**Figure 3:**
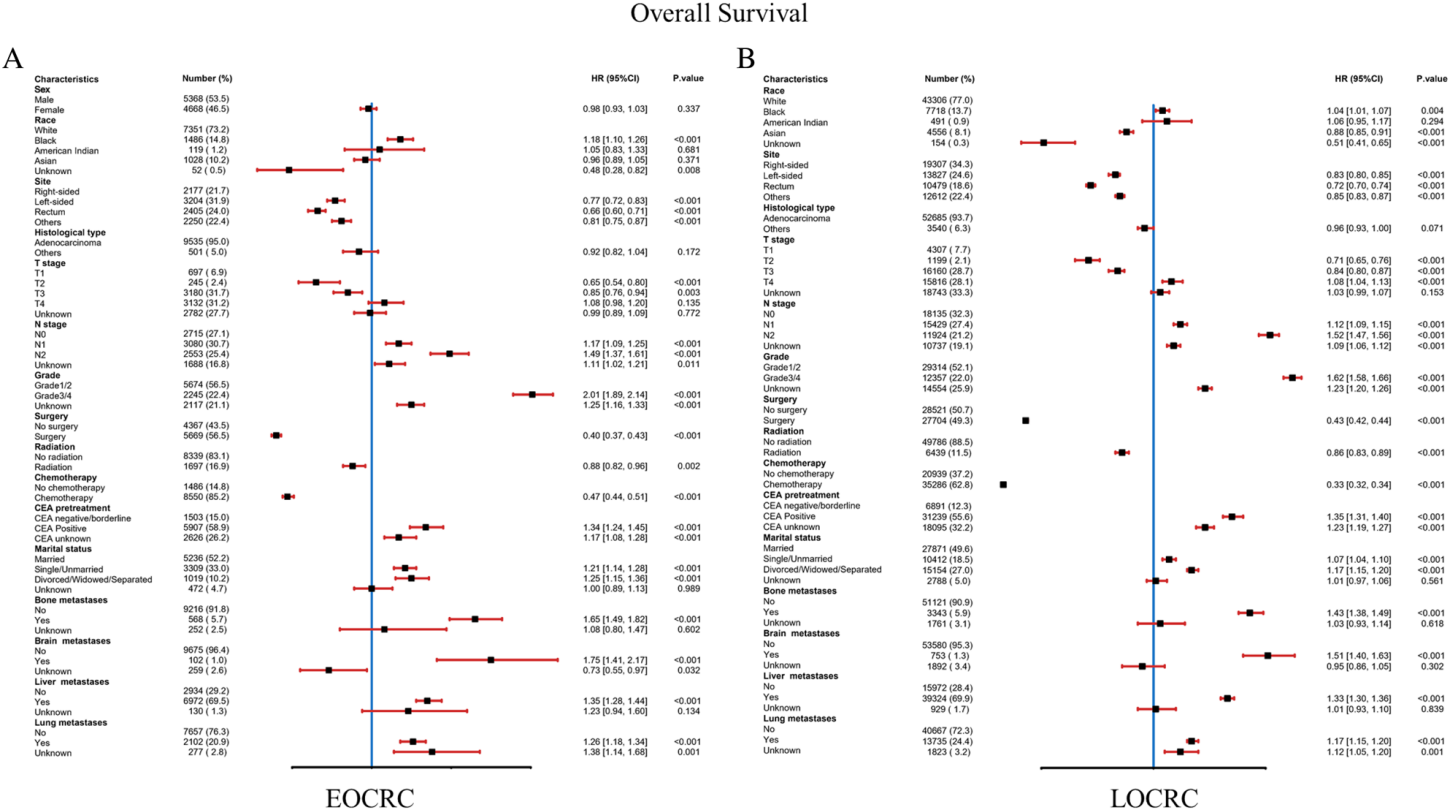
Forest plot of multivariate Cox regression analysis for OS in early-onset colorectal cancer chemotherapy (EOCRC) and late-onset colorectal cancer (LOCRC).

The OS of early-onset colorectal cancer (EOCRC) and late-onset colorectal cancer (LOCRC) was calculated using the Kaplan–Meier curves. The results indicated significant differences in OS between EOCRC and LOCRC (*p* < 0.0001) (Fig. 2). The median OS for EOCRC was 18 months (95% CI 8–33 months), while for LOCRC it was 10 months (95% CI 3–24 months). A univariable Cox proportional hazards regression analysis was performed in the EOCRC population (Table 2), considering all baseline information related to patient's OS, such as gender, primary site, race, histology, T stage, N stage, grade, surgery, radiation, chemotherapy, CEA, marital status, bone metastases, brain metastases, liver metastases, and lung metastases. These variables were also included in the subsequent multivariable Cox proportional hazards regression analysis. In the multivariate analysis, primary site, race, histology, T stage, N stage, grade, surgery, radiation, chemotherapy, CEA, marital status, bone metastases, brain metastases, liver metastases, and lung metastases were identified as independent risk or protective factors. When performing univariable Cox proportional hazards regression analysis in the LOCRC population (Table 3), primary site, race, histology, T stage, N stage, grade, surgery, radiation, chemotherapy, CEA, marital status, bone metastases, brain metastases, liver metastases, and lung metastases were related to OS. These factors were adjusted in subsequent multivariate analyses. primary site, race, histology, T stage, N stage, grade, surgery, radiation, chemotherapy, CEA, marital status, bone metastases, brain metastases, liver metastases, and lung metastases have been confirmed to be independent risk or protective factors. Forest plots of multivariate Cox regression analysis for OS in early-onset colorectal cancer (EOCRC) and late-onset colorectal cancer (LOCRC) were also conducted (Fig. 3).

**Table 2:** Univariable and multivariate Cox regression analysis based on all variables for overall survival of EOCRC patients.

Variable	Univariable		Multivariable	
	HR (95%CI)	*p*	HR (95%CI)	*p*
Gender				
Male	[Reference]		[Reference]	
Female	0.902(0.859–0.947)	< 0.001	0.976(0.929–1.026)	0.337
Race				
White	[Reference]		[Reference]	
Black	1.260(1.178–1.347)	< 0.001	1.176(1.098–1.260)	< 0.001
American Indian	0.935(0.741–1.180)	0.571	1.050(0.831–1.327)	0.681
Asian	0.981(0.904–1.065)	0.648	0.963(0.886–1.046)	0.371
Unknown	0.538(0.312–0.927)	< 0.05	0.4759(0.276–0.821)	< 0.01
Primary site				
Right	[Reference]		[Reference]	
Left	0.728(0.681–0.778)	< 0.001	0.773(0.722–0.826)	< 0.001
Rectum	0.736(0.686–0.791)	< 0.001	0.656(0.604–0.712)	< 0.001
Others	0.749(0.697–0.805)	< 0.001	0.808(0.750–0.871)	< 0.001
Histological type				
Adenocarcinoma	[Reference]		[Reference]	
Others	1.362(1.225–1.513)	< 0.001	0.924(0.824–1.035)	0.172
T				
T1	[Reference]		[Reference]	
T2	0.463(0.381–0.561)	< 0.001	0.655(0.537–0.798)	< 0.001
T3	0.548(0.497–0.604)	< 0.001	0.850(0.765–0.950)	< 0.01
T4	0.753(0.684–0.828)	< 0.001	1.083(0.976–1.202)	0.135
Unknown	1.144(1.039–1.260)	< 0.01	0.986(0.893–1.088)	0.772
N				
N0	[Reference]		[Reference]	
N1	0.965(0.905–1.029)	0.274	1.166(1.090–1.248)	< 0.001
N2	1.031(0.964–1.102)	0.379	1.485(1.374–1.605)	< 0.001
Unknown	1.622(1.499–1.754)	< 0.001	1.113(1.025–1.209)	< 0.05
Grade				
1/2	[Reference]		[Reference]	
3/4	1.954(1.843–2.072)	< 0.001	2.013(1.893–2.141)	< 0.001
Unknown	1.882(1.769–2.003)	< 0.001	1.246(1.164–1.334)	< 0.001
Surgery				
No	[Reference]		[Reference]	
Yes	0.416(0.395–0.437)	< 0.001	0.396(0.368–0.425)	< 0.001
Radiation				
No	[Reference]		[Reference]	
Yes	0.800(0.748–0.855)	< 0.001	0.885(0.819–0.956)	< 0.01
Chemotherapy				
No	[Reference]		[Reference]	
Yes	0.487(0.456–0.519)	< 0.001	0.475(0.443–0.509)	< 0.001
CEA				
Negative/Borderline	[Reference]		[Reference]	
Positive	1.603(1.485–1.730)	< 0.001	1.345(1.244–1.454)	< 0.001
Unknown	1.451(1.335–1.578)	< 0.001	1.174(1.077–1.280)	< 0.001
Marital status				
Married	[Reference]		[Reference]	
Single/Unmarried	1.291(1.223–1.363)	< 0.001	1.208(1.142–1.277)	< 0.001
Divorced/Widowed/Separated	1.251(1.154–1.357)	< 0.001	1.253(1.154–1.360)	< 0.001
Unknown	1.098(0.977–1.234)	0.116	1.001(0.890–1.126)	0.989
Bone metastases				
No	[Reference]		[Reference]	
Yes	2.338(2.125–2.572)	< 0.001	1.647(1.489–1.822)	< 0.001
Unknown	1.351(1.172–1.559)	< 0.001	1.085(0.799–1.472)	0.602
Brain metastases				
No	[Reference]		[Reference]	
Yes	2.289(1.855–2.826)	< 0.001	1.746(1.405–2.171)	< 0.001
Unknown	1.261(1.094–1.454)	< 0.01	0.731(0.549–0.973)	< 0.05
Liver metastases				
No	[Reference]		[Reference]	
Yes	1.325(1.254–1.401)	< 0.001	1.353(1.276–1.436)	< 0.001
Unknown	1.443(1.175–1.773)	< 0.001	1.226(0.939–1.601)	0.134
Lung metastases				
No	[Reference]		[Reference]	
Yes	1.495(1.410–1.585)	< 0.001	1.259(1.184–1.339)	< 0.001
Unknown	1.502(1.309–1.723)	< 0.001	1.381(1.136–1.679)	< 0.01

**Table 3:** Univariable and multivariate Cox regression analysis based on all variables for overall survival of LOCRC patients.

Variable	Univariable		Multivariable	
	HR (95%CI)	*p*	HR (95%CI)	*p*
Gender				
Male	[Reference]		[Reference]	
Female	1.006(0.987–1.025)	0.526		
Race				
White	[Reference]		[Reference]	
Black	1.100(1.0707–1.130)	< 0.001	1.041(1.013–1.070)	< 0.01
American Indian	1.064(0.963–1.176)	0.225	1.055(0.955–1.166)	0.294
Asian	0.902(0.871–0.935)	< 0.001	0.879(0.848–0.911)	< 0.001
Unknown	0.696(0.551–0.880)	< 0.01	0.513(0.405–0.648)	< 0.001
Primary site				
Right	[Reference]		[Reference]	
Left	0.789(0.770–0.809)	< 0.001	0.826(0.805–0.847)	< 0.001
Rectum	0.773(0.753–0.795)	< 0.001	0.719(0.698–0.742)	< 0.001
Others	0.914(0.891–0.937)	< 0.001	0.852(0.829–0.874)	< 0.001
Histological type				
Adenocarcinoma	[Reference]		[Reference]	
Others	1.357(1.307–1.409)	< 0.001	0.964(0.927–1.003)	0.071
T				
T1	[Reference]		[Reference]	
T2	0.485(0.449–0.524)	< 0.001	0.707(0.653–0.765)	< 0.001
T3	0.565(0.544–0.586)	< 0.001	0.838(0.804–0.874)	< 0.001
T4	0.776(0.747–0.805)	< 0.001	1.080(1.036–1.125)	< 0.001
Unknown	1.203(1.160–1.247)	< 0.001	1.028(0.990–1.066)	0.153
N				
N0	[Reference]		[Reference]	
N1	0.831(0.811–0.852)	< 0.001	1.121(1.092–1.151)	< 0.001
N2	0.945(0.921–0.970)	< 0.001	1.516(1.470–1.564)	< 0.001
Unknown	1.505(1.464–1.547)	< 0.001	1.088(1.057–1.120)	< 0.001
Grade				
1/2	[Reference]		[Reference]	
3/4	1.569(1.533–1.607)	< 0.001	1.615(1.576–1.655)	< 0.001
Unknown	1.807(1.766–1.848)	< 0.001	1.228(1.197–1.259)	< 0.001
Surgery				
No	[Reference]		[Reference]	
Yes	0.472(0.463–0.482)	< 0.001	0.430(0.417–0.443)	< 0.001
Radiation				
No	[Reference]		[Reference]	
Yes	0.700(0.679–0.722)	< 0.001	0.860(0.831–0.890)	< 0.001
Chemotherapy				
No	[Reference]		[Reference]	
Yes	0.349(0.342–0.356)	< 0.001	0.330(0.324–0.337)	< 0.001
CEA				
Negative/Borderline	[Reference]		[Reference]	
Positive	1.491(1.445–1.538)	< 0.001	1.354(1.311–1.398)	< 0.001
Unknown	1.561(1.510–1.614)	< 0.001	1.229(1.188–1.271)	< 0.001
Marital status				
Married	[Reference]		[Reference]	
Single/Unmarried	1.200(1.169–1.231)	< 0.001	1.072(1.044–1.100)	< 0.001
Divorced/Widowed/Separated	1.376(1.346–1.407)	< 0.001	1.173(1.147–1.200)	< 0.001
Unknown	1.179(1.128–1.231)	< 0.001	1.013(0.969–1.059)	0.561
Bone metastases				
No	[reference]		[reference]	
Yes	0.564(0.543–0.585)	< 0.001	1.432(1.377–1.489)	< 0.001
Unknown	0.842(0.792–0.895)	< 0.001	1.027(0.926–1.138)	0.618
Brain metastases				
No	[Reference]		[Reference]	
Yes	1.947(1.803–2.101)	< 0.001	1.511(1.396–1.635)	< 0.001
Unknown	1.473(1.403–1.546)	< 0.001	0.950(0.862–1.047)	0.302
Liver metastases				
No	[Reference]		[Reference]	
Yes	1.246(1.219–1.272)	< 0.001	1.333(1.304–1.364)	< 0.001
Unknown	1.428(1.330–1.534)	< 0.001	1.009(0.926–1.099)	0.839
Lung metastases				
No	[Reference]		[Reference]	
Yes	1.300(1.272–1.328)	< 0.001	1.175(1.149–1.202)	< 0.001
Unknown	1.564(1.488–1.644)	< 0.001	1.125(1.050–1.205)	< 0.001

### Survival outcomes between EOCRC and LOCRC patients stratified by primary site, the location of metastasis

**Figure 4:**
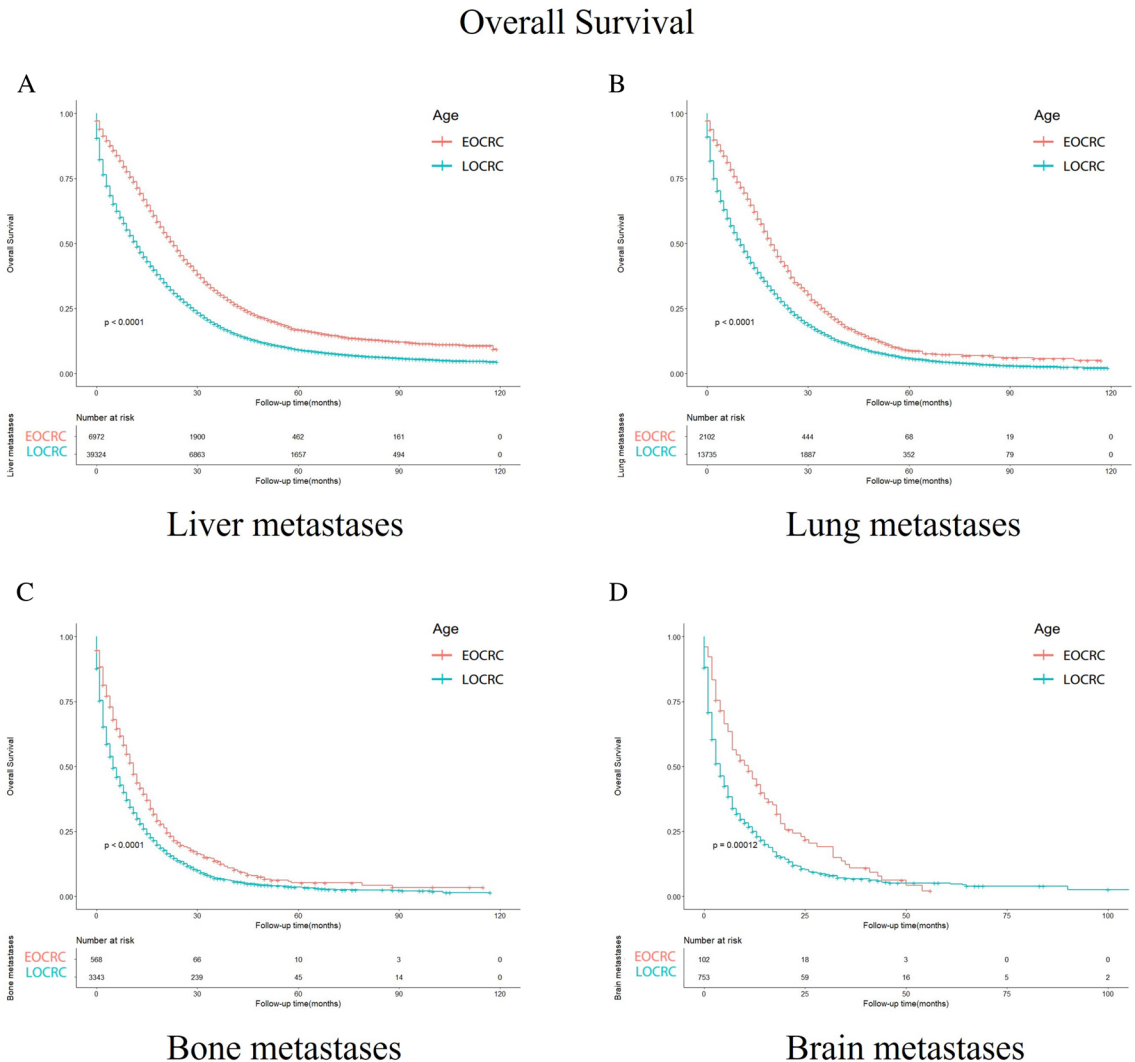
Kaplan–Meier-curves for overall survival in EOCRC and LOCRC patients with different metastasis sites. Life tables for patients at risk are given below each plot. (**A**) Liver metastases. (**B**) Lung metastases. (**C**) Bone metastases. (**D**) Brain metastases.

**Figure 5:**
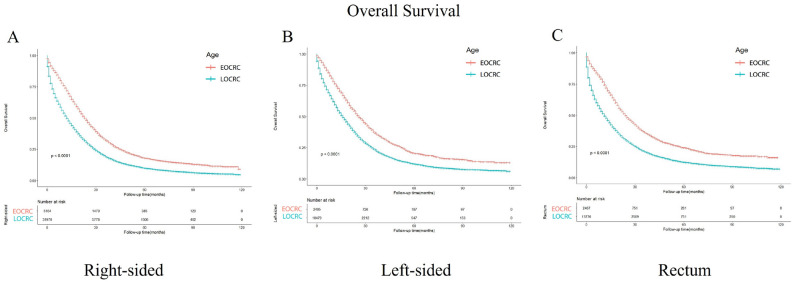
Kaplan–Meier-curves for overall survival in EOCRC and LOCRC patients with different tumor primary sites. Life tables for patients at risk are given below each plot. (**A**) Right-sided. (**B**) Left-sided. (**C**) Rectum.

In the subgroups divided according to the metastasis site (Fig. 4), the OS of EOCRC patients with liver, lung, brain and bone metastases is better than that of LOCRC patients (*p* < 0.001, *p* < 0.001, *p* < 0.001, and *p* < 0.001). In terms of cancer primary location, the survival outcomes of all the subgroups show that EOCRC patients’ OS is better than LOCRC patients’ OS (*p* < 0.001) (Fig. 5).

### Survival outcomes between EOCRC and LOCRC patients stratified by treatment modalities

**Figure 6:**
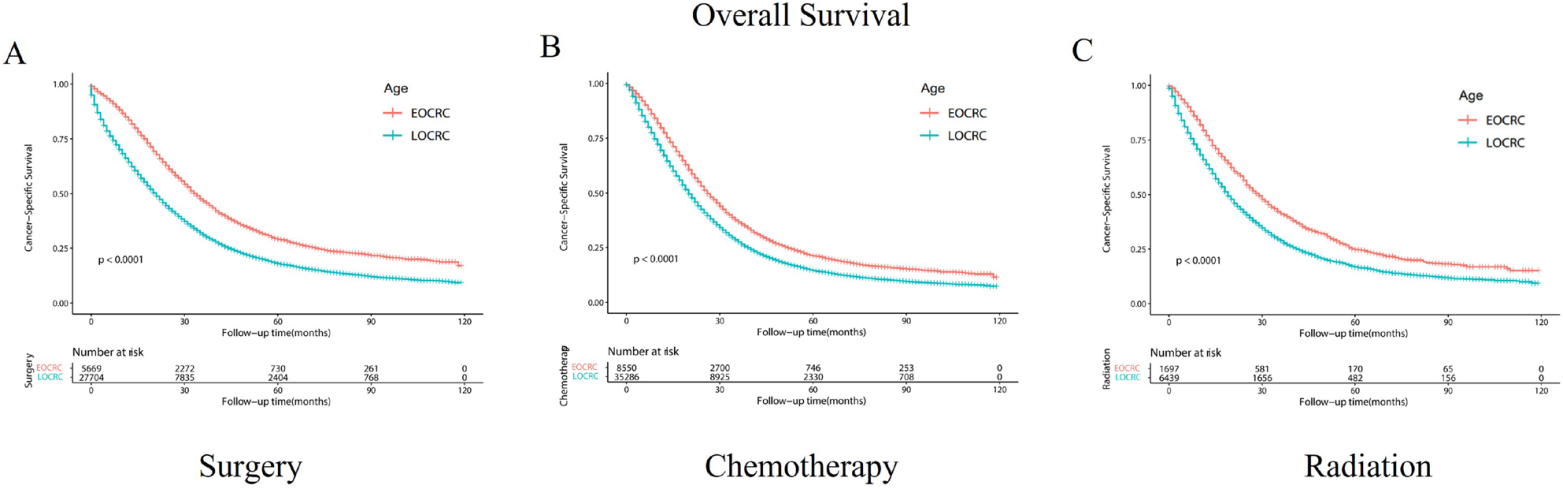
Kaplan–Meier-curves for overall survival in EOCRC and LOCRC patients with different treatment modalities. Life tables for patients at risk are given below each plot. (**A**) Surgery. (**B**) Chemotherapy. (**C**) Radiation.

Subsequently, we conducted a comparison of the overall survival among patients diagnosed with EOCRC and LOCRC who underwent various treatment modalities. The findings indicated that within the subgroups of surgery, chemotherapy, and radiotherapy, the overall survival was notably higher among EOCRC patients in comparison to those with LOCRC (*p* < 0.0001) (Fig. 6).

### Survival outcomes between male and female patients stratified by age

**Figure 7:**
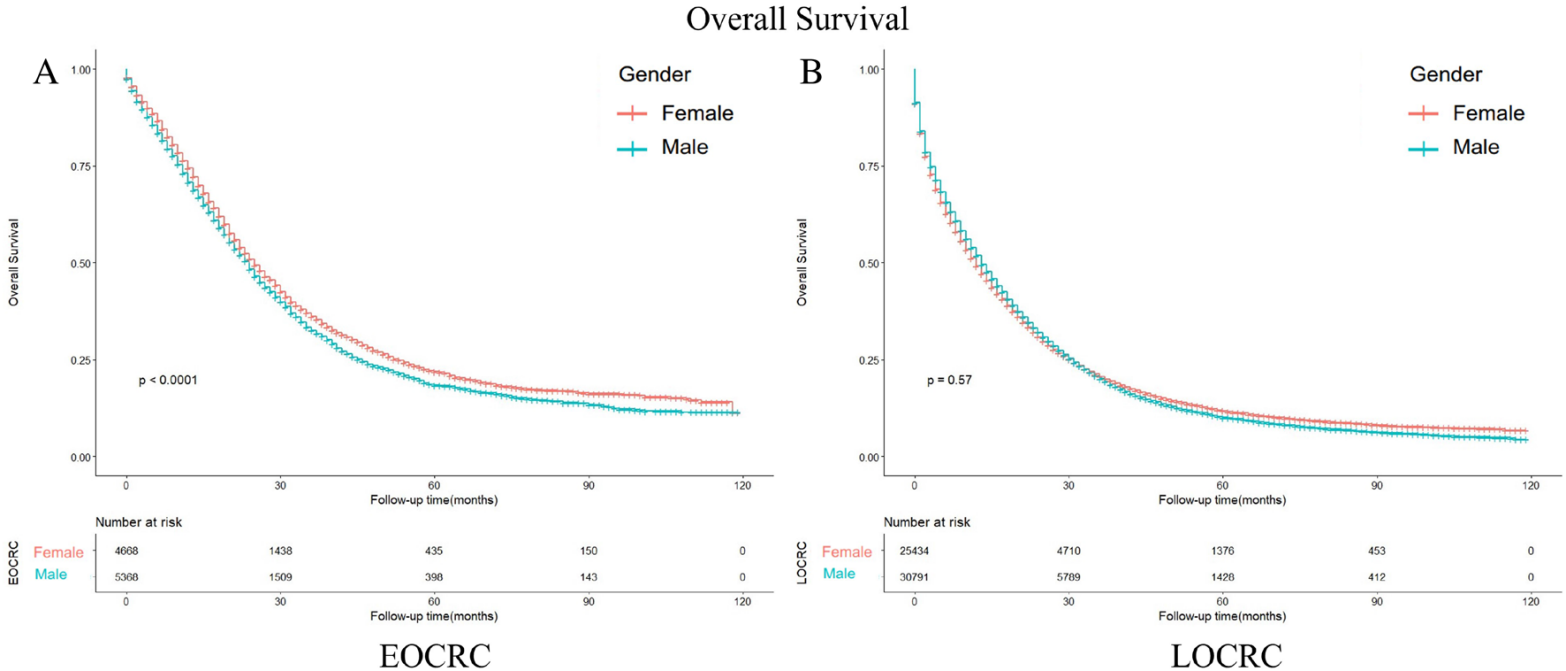
Kaplan–Meier-curves for overall survival in male and female patients with different age. Life tables for patients at risk are given below each plot. (**A**) EOCRC patients. (**B**) LOCRC patients.

In univariate analysis, we observed differences in OS based on gender for early-onset colorectal cancer, but no such differences for late-onset colorectal cancer (Tables 2,3). We utilized Kaplan–Meier curves to do further analysis. In gender-divided subgroups, the OS shows differences between male EOCRC patients and female EOCRC patients (*p* < 0.001) (Fig. 7A). When performing analysis on male and female LOCRC patients, these differences disappear (*p* = 0.57) (Fig. 7B).

## Discussion

In the United States, there has been a decline in colorectal cancer incidence and mortality rates due to increased colorectal cancer screening rates^19^. However, there is a concerning increase in early-onset colorectal cancer, which is also being observed globally^20^. This rise may be attributed to changes in eating habits associated with global economic development, such as increased consumption of sugary drinks, red meat, and processed meat products. Additionally, factors such as obesity, sedentary lifestyles, alcohol consumption, and the increased incidence of inflammatory bowel disease may contribute to this trend^21–24^. Despite these observations, our understanding of early-onset colorectal cancer remains limited, and further research is necessary to gain insights into this disease. Successful implementation of primary or secondary prevention strategies for early-onset colorectal cancer could have a significant impact on life expectancy in the future. Metastasis is the leading cause of mortality in colorectal cancer and significantly affects the prognosis of patients^25–29^. Distal organ metastasis is also a characteristic of young-onset cases. Therefore, we compared metastatic early-onset colorectal cancer with metastatic late-onset colorectal cancer to identify differences. Previous studies have explored the influence of various prognostic factors on patients with stage IV colorectal cancer^28^. Some demographic and clinicopathological variables have been identified as independent prognostic factors, including age at diagnosis, tumor location and size, and histological differentiation. However, due to the limited scale of these studies^11,12^, they do not fully capture the prognosis of stage IV colorectal cancer patients nor allow for a comprehensive comparison between early-onset and late-onset cases. This hinders our ability to provide more precise treatment for colorectal cancer patients based on age.

Comparing the survival of EOCRC and LOCRC, we found that the survival rate of EOCRC is approximately 8 months higher than that of LOCRC in metastatic colorectal cancer. Our research project has a larger sample size (66,261 vs. 254) compared to other studies^11^. Baseline data indicate that white individuals have a lower rate of early-onset colorectal cancer, which may be attributed to the quality of medical care they receive^30^. Younger patients showed a higher prevalence of mucus histology, consistent with another study^31^. The proportion of T3/4 patients in early-onset colorectal cancer patients is significantly higher than in late-onset colorectal cancer patients, indicating that the tumor size and extent are greater in early-onset cases. Similarly, N stage reveals that early-onset colorectal cancer patients have higher N1 or N2 ratios, indicating a significantly greater degree of lymph node involvement compared to late-onset cases. Despite these differences, early-onset colorectal cancer still has a better prognosis. This may be attributed to differences in treatment options, as early-onset cases often receive more aggressive treatment including surgery, radiotherapy, and chemotherapy^32,33^. Although some studies suggest that more aggressive treatment for early-onset colorectal cancer does not result in improved survival^34–36^, our results support the need for such treatment in younger patients with colorectal cancer.

In the subgroup analysis based on the location of metastasis, we examined the four most common metastatic organs: liver, lung, brain, and bone. Among these four metastases, the survival rate of early-onset colorectal cancer differed significantly from that of late-onset colorectal cancer. The prognosis for early-onset colorectal cancer was better than that for late-onset colorectal cancer (*p* < 0.001). Regarding specific metastases, liver metastasis was the most frequent, followed by lung metastasis, bone metastasis, and finally brain metastasis, which was the least common. These findings align with previous studies that indicate the liver as the most common site of metastatic disease in colorectal cancer patients^25^. In terms of location-related prognosis, for early-onset colorectal cancer, liver metastasis had the most favorable prognosis, with a median survival time of 17 months (95% CI 7–31 months), followed by lung metastasis at 15 months (95% CI 6–26 months), brain metastasis at 9.5 months (95% CI 7–31 months 3.25–18.75), and bone metastasis with the worst prognosis at 9 months (95% CI 7–31 months 3.75–17). In late-onset colorectal cancer, liver metastasis still had the best prognosis, with a median survival time of 9 months (95% CI 2–23 months). Lung metastasis had the second-best prognosis, with a median survival time of 8 months (95% CI 2–20 months), followed by bone metastasis at 5 months (95% CI 1–13 months), and brain metastasis with the worst prognosis at 3 months (95% CI 1–11 months). Previous studies have indicated that the 5-year survival rate of colorectal cancer (CRC) patients with brain metastases is lower than that of patients with liver metastases^37^. The different prognosis between early-onset colorectal cancer and late-onset colorectal cancer suggests the need for different treatment options based on age, which could help improve patient survival rates.

In subgroup analysis based on tumor primary location, our study found that early-onset colorectal cancer is associated with better survival compared to late-onset colorectal cancer, regardless of whether the primary location is on the left side, right side, or rectum. Among these three locations, the left side exhibits the highest survival rate, followed by the rectum, while the right side shows the lowest survival rate. These findings align with numerous previous studies^38^, as the right colon is generally linked to worse survival due to higher BRAF mutations and higher microsatellite instability^39–42^. Furthermore, aside from location-related differences in molecular biology, some studies suggest that different locations in the colon, including the rectum, have distinct embryonic origins^43^. Additionally, alterations in microbiota, stool composition, enzymes, and metabolites along the intestine may also contribute to variations in the colon^44,45^. The reasons behind the differences in survival rates among the left side, right side or the rectum, are multifactorial. Clinical studies have also demonstrated that patients with colorectal cancer benefit more from first-line treatment if the primary site is on the left side, while patients with the primary site on the right experience minimal effects. Our study suggests that for patients with metastatic colorectal cancer, the preference for the left-side colon over the right-side colon remains consistent in both early-onset and late-onset cases, with early-onset colorectal cancer having a more favorable prognosis compared to late-onset colorectal cancer.

We conducted a multivariable Cox proportional hazards regression analysis for both early-onset and late-onset colorectal cancer to assess overall survival (OS). For early-onset colorectal cancer, significant prognostic factors for OS include primary site, race, histology, T stage, N stage, grade, surgery, radiation, chemotherapy, CEA, marital status, bone metastases, brain metastases, liver metastases, and lung metastases. Similarly, for late-onset colorectal cancer, the significant prognostic factors for OS align with those for early-onset colorectal cancer.

However, in univariate analysis, we observed differences in OS based on gender for early-onset colorectal cancer, but no such differences for late-onset colorectal cancer. We suspect that the survival difference between men and women in early-onset colorectal cancer may be influenced by age. To investigate this further, we performed a Kaplan–Meier curves using age as a reference and found that among patients with early-onset colorectal cancer, female patients had a significantly better prognosis than male patients, whereas this difference in prognosis between men and women disappeared in patients with late-onset colorectal cancer. Previous studies have consistently found that male sex is an independent risk factor for overall survival (OS) in colorectal cancer. This association may be attributed to differences in men's lifestyle, such as higher levels of financial and mental stress, as well as higher rates of smoking and alcohol consumption^46^. Furthermore, some studies suggest that the disparity in colorectal cancer incidence between genders could be linked to male-specific genes found on the Y chromosome^47^. However, despite these explanations, it remains challenging to fully elucidate the age-related differences observed between men and women. Therefore, we hypothesize that the inconsistent age-related prognosis of metastatic early-onset colorectal cancer in men and women could be influenced by sex hormones. Notably, women experience menopause around the age of 50, which coincides with this age-related difference. Estrogen, a crucial sex hormone^48,49^, exerts various biological effects and has been shown in previous studies to inhibit colorectal cancer by altering the ratio of ERα to ERβ^50^. Additionally, estrogen may exert a protective role by influencing MSI status and gene methylation^51^. Moreover, clinical investigations have demonstrated that estrogen replacement therapy in postmenopausal women can reduce the risk of colorectal cancer^52^. Based on these findings, we propose the implementation of gender-specific prevention and treatment strategies for early-onset colorectal cancer.

The strengths of this article are as follows: Firstly, this is the first article to compare metastatic early-onset colorectal cancer with late-onset colorectal cancer. We conducted a detailed and comprehensive analysis to better understand the differences between these two types of colorectal cancer. Secondly, our study has a larger sample size compared to other articles that have studied early-onset colorectal cancer. This helps to eliminate some of the biases that can arise from small sample sizes. Moreover, we first discover the sex differences between EOCRC and LOCRC, which is very meaningful for precision medicine based on sex. Lastly, our findings serve as a valuable supplement and confirmation of previous studies.

However, it is important to acknowledge the limitations of our study. Firstly, due to the nature of the SEER database, our study can only establish observational correlations and cannot analyze causal relationships like MR studies. Secondly, some important factors may be neglected due to unavailable data in the SEER database, such as chemotherapy regimen, targeted therapy, bisphosphonates usage, etc. Therefore, RCT studies should be designed to further investigate and confirm our conclusions in the future.

## Conclusion

Metastatic early-onset colorectal cancer patients have longer survival time than late-onset colorectal cancer patients. Tumor primary location, the location of metastasis and treatment modalities affect the survival outcomes between these two groups of patients. The sex differences in survival of metastatic colorectal cancer patients are associated with patients’ age. These findings contribute to a better understanding of the differences between metastatic early-onset colorectal cancer and late-onset colorectal cancer, and can help inform the development of more precise treatment guidelines to improve prognosis.

## Data Availability

Informed patient consent was not required for data obtained from SEER, as cancer is a publicly reportable disease in every state in the USA. The datasets generated and analyzed during the current study are available in SEER database. [https://seer.cancer.gov/].
